# Bee Products: An Emblematic Example of Underutilized Sources of Bioactive Compounds

**DOI:** 10.1021/acs.jafc.1c05822

**Published:** 2022-01-02

**Authors:** Francesca Giampieri, Jose Luis Quiles, Danila Cianciosi, Tamara Yuliett Forbes-Hernández, Francisco Josè Orantes-Bermejo, José Miguel Alvarez-Suarez, Maurizio Battino

**Affiliations:** ‡ Department of Biochemistry, Faculty of Sciences, King Abdulaziz University, Jeddah 21589, Saudi Arabia; § Research Group on Food, Nutritional Biochemistry and Health, Universidad Europea del Atlántico, 39011 Santander, Spain; ∥ Department of Clinical Sciences, Polytechnic University of Marche, 60131 Ancona, Italy; ⊥ Department of Physiology, Institute of Nutrition and Food Technology ‘‘José Mataix”, Biomedical Research Centre, University of Granada, 1800 Granada, Spain; # Department of Analytical and Food Chemistry, CITACA, CACTI, University of Vigo, 36310 Vigo, Spain; ∇ Apinevada Analytical Laboratory of Bee Products, Barrancos s/n, Lanjarón, Granada 18420, Spain; ○ Departamento de Ingeniería en Alimentos, Colegio de Ciencias e Ingenierías, Universidad San Francisco de Quito, Quito 170157, Ecuador; ◆ King Fahd Medical Research Center, King Abdulaziz University, Jeddah 21589, Saudi Arabia; ¶ International Joint Research Laboratory of Intelligent Agriculture and Agri-products Processing, Jiangsu University, Zhenjiang, Jiangsu 212013, People’s Republic of China; α Instituto de Investigaciones en Biomedicina iBioMed, Universidad San Francisco de Quito, Quito 170157, Ecuador

**Keywords:** honeybee byproducts, bee pollen, propolis, bee bread, royal jelly, beeswax

## Abstract

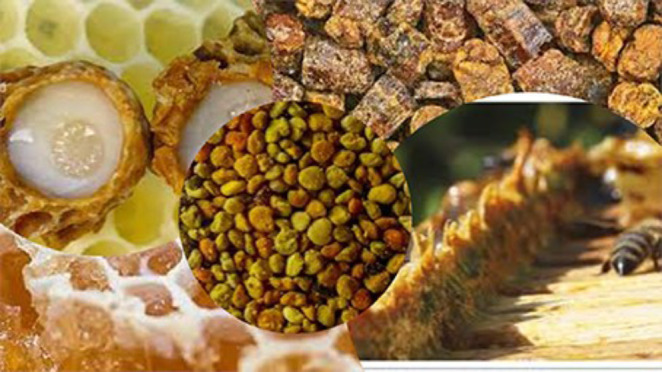

Beside honey, honeybees (*Apis mellifera* L.) are able to produce many byproducts, including bee pollen, propolis, bee bread, royal jelly, and beeswax. Even if the medicinal properties of these byproducts have been recognized for thousands of years by the ancient civilizations, in the modern era, they have a limited use, essentially as nutritional supplements or health products. However, these natural products are excellent sources of bioactive compounds, macro- and micronutrients, that, in a synergistic way, confer multiple biological activities to these byproducts, such as, for example, antimicrobial, antioxidant, and anti-inflammatory properties. This work aims to update the chemical and phytochemical composition of bee pollen, propolis, bee bread, royal jelly, and beeswax and to summarize the main effects exerted by these byproducts on human health, from the anticancer and immune-modulatory activities to the antidiabetic, hypolipidemic, hypotensive, and anti-allergic properties.

## 1 Introduction

Bees are a large group of social insects belonging to the Apidae family, which includes honey or domestic bees, stingless bees, and other specific groups. In particular, honeybees (western *Apis mellifera*, distributed mainly in Europe, America, Africa, and Asia, and eastern *Apis cerana*, native to Southeast Asia) are the two best known domesticated species used in modern beekeeping. These honeybees produce and store in the beehives several products that are potentially beneficial for human health. Undoubtedly, the most famous and widely appreciated honeybee product is honey, a complex mixture of nutrients and bioactive compounds with multiple biological activities.^1−7^ However, besides honey, bees are also able to make several other types of products, including bee pollen (BP), propolis, bee bread (BB), royal jelly (RJ), and beeswax (BW). These products may derive from pollen grains, nectars, and other plant materials alone or mixed with the bee salivary gland secretions and plant secretions.^8^ In the last years, they have also attracted the interest of the scientific community worldwide: numerous studies have found beneficial effects exerted by these natural products on human health, highlighting their potential use as active pharmaceutical ingredients.^8^ In addition, some efforts have also been done to introduce these products in clinical practice, but these attempts have almost failed because of their high chemical, nutritional, and phytochemical variabilities that depend upon several parameters, including, for example, the honeybee varieties, the plant, the geographical area, and the seasons, which makes the medicinal standardization difficult to perform. Despite all of this evidence, currently, these products have few applications, mainly in the nutraceutical and food industries as dietary supplements. With this background, this review aims to present the nutritional and phytochemical contents of BP, propolis, BB, RJ, and beeswax and to summarize the biological properties played by these products, given the urgent need to find new remedies against the most common human pathologies, such as metabolic and cardiovascular diseases.

## 2 Bee Pollen

Bee pollen (BP) is produced as a result of the collection of pollen grains by bees that are agglutinated using salivary secretions, nectar, and/or honey to form a granule of ∼1.4–4 mm in size, which is stored in the alveoli of the hive until consumption. A colony of bees can collect between 50 and 250 g of pollen per day, totaling 15–40 kg per year. The main function of pollen is to serve as a source of nutrients for the colony, thus guaranteeing its development and maintenance. From a compositional point of view, BP is often a valuable source of proteins, essential amino acids, vitamins, and fatty acids as well as other compounds that, although they lack a nutritional function, exhibit an important functional character, such as pigments (carotenoids) and polyphenols, which can act as potent antioxidants.^9,10^ This composition is influenced by several factors, for instance, the plant species, geographical region, and season of the year in which the pollen is collected. Thus, these constituents vary in content levels.^11^ Despite this, pollen remains a valuable source of essential nutrients and non-nutritive compounds, to the point that is often regarded as “the world’s best food product”.^12^

BP is an excellent source of carbohydrates, its main component. In fact, carbohydrates make up two-thirds of its dry weight^13,14^ and are incorporated from honey or nectar used for pollen pellet formation.^15^ Therefore, the type of plant, together with climatic conditions and geographical origin, plays a fundamental role in the carbohydrate content.^11^ For these reasons, data collected on BP samples of different botanical and geographical origins show a huge variation (Table 1). Monosaccharides are the main sugars present, accounting for about 94% of total sugars,^9^ with a large amount of reducing sugars, making it distinct from plant pollen.^13^ Fructose and glucose, with the ratio varying between 1.20 and 1.50, are the most abundant sugars, followed by sucrose, maltose, and other disaccharides, such as sucrose, turanose, erlose, maltose, and trehalose.^16^ A total of 3–4% of BP is cellulose, which is the main component of the layers of pollen grains, and its presence significantly affects the digestibility of BP.^9^

**Table 1:** Bee Pollen Composition from Different Geographical and Botanical Origins^a^ ^9−14,17,18^

proximate	content (min–max)		
moisture (%)	1.50–13.80		
dietary fiber (%)	0.15–30.00		
proteins (%)	2.50–62.00		
ash (%)	0.50–6.50		
lipids (%)	0.41–24.40		
carbohydrates (%)	18.50–82.80		
sugars (g/100 g)			
glucose	2.77–28.49		
fructose	4.90–33.48		
sucrose	0.05–9.02		
maltose	0.16–6.03		
isomaltose	0.10–0.60		
raffinose	0.10–0.20		
trehalose	0.10–0.40		
erlose	0.10–0.30		
polyphenols			
total phenolic content (mg of GAE/g)	0.69–213.00		
total flavonoid content (mg of QE/g)	1.82–107.00		
fatty acids (g/100 g)			
C4:0 butyric acid	traces–0.26	C18:3 α-linolenic acid	0.1–56.90
C6:0 caproic acid	traces–4.53	C18:3 γ-linolenic acid	traces–41.99
C8:0 caprylic acid	traces–6.34	C20:0 arachidic acid	traces–6.88
C10:0 capric acid	traces–14.98	C20:1 eicosenoic acid	0.0073–3.35
C11:0 undecanoic acid	traces–1.70	C21:1 heneicosenoic acid	traces–3.92
C12:0 lauric acid	traces–8.51	C20:2 eicosadienoic acid	traces–4.27
C14:0 myristic acid	traces–20.70	C20:3 dihomo-γ-linolenic acid	traces–54.01
C15:0 pentadecanoic acid	traces–29.97	C20:3 eicosatrienoic acid	traces–18.36
C16:0 palmitic acid	traces–64.38	C20:4 arachidonic acid	traces–2.40
C16:1 palmitoleic acid	traces–4.39	C22:0 behenic acid	traces–17.89
C17:0 heptadecanoic acid	0.07–25.60	C22:1 erucic acid	0.0038–1.40
C17:1 heptadecenoic acid	traces–6.61	C22:6 docosahexaenoic acid	traces–1.29
C18:0 stearic acid	0.0027–8.52	C23:0 tricosanoic acid	0.09–5.39
C18:1 *cis*-oleic acid	1.33–20.61	C20:5 eicosapentaenoic acid	0.35–3.36
C18:2 *trans*-linoelaidic acid	0.22–12.62	C24:0 lignoceric acid	traces–1.59
C18:2 *cis*-linoleic acid	0.0085–53.92	C24:1 nervonic acid	traces–0.97
total amino acids (g/100 g)			
essential amino acids		non-essential amino acids	
arginine	0.03–2.58	alanine	0.09–2.33
histidine	0.07–4.49	aspartic acid	0.21–3.23
isoleucine	0.01–1.60	cysteine	0.07–0.30
leucine	0.06–2.47	glutamic acid	0.13–3.03
lysine	0.03–3.71	glycine	0.04–1.84
methionine	0.01–0.62	proline	0.04–19.8
phenylalanine	0.03–2.72	serine	0.23–1.33
threonine	0.02–5.58	tyrosine	0.03–3.76
tryptophan	0.05–14.80	asparagine	0.08–0.57
valine	0.03–1.57	glutamine	0.02–1.42
vitamins (mg/100 g)			
fat soluble		water soluble	
α-carotene	0.33–32.47	thiamine (vitamin B_1_)	0.20–1.30
β-carotene	0.08–19.89	riboflavin (vitamin B_2_)	0.40–2.56
γ-carotene	5.38–12.87	niacin (vitamin B_3_)	1.30–15.34
ξ-carotene	4.49–11.58	nicotinamide (vitamin B_3_)	0.51–12.10
ε-carotene	5.80–12.39	pantothenic acid (vitamin B_5_)	0.50–2.00
lutein	4.45–47.63	pyridoxine (vitamin B_6_)	0.10–3.80
β-cryptoxanthin	0.13–8.54	biotin (vitamin B_7_)	0.05–0.07
isocryptoxanthin	3.11–8.05	folic acid	0.30–1.00
isozeaxanthin	3.80–26.54	vitamin C	6.03–79.70
lactucaxanthin	3.17–9.80		
neoxanthin	4.45–7.22		
violaxanthin	4.83–10.60		
antheraxanthin	4.01–9.15		
astaxanthin	3.69–9.01		
canthaxanthin	4.50–9.01		
tocopherols (vitamin E)	0.46–9.57		
minerals (mg/kg)			
macrominerals		microminerals	
potassium (K)	3.60–13366.60	zinc (Zn)	0.10–340.00
calcium (Ca)	1.09–5752.19	iron (Fe)	2.60–1180.00
phosphorus (P)	234.40–9687.00	manganese (Mn)	0.10–430.00
magnesium (Mg)	44.0–4680.53	copper (Cu)	3.73–42.00
sodium (Na)	4.95–8350.27	selenium (Se)	0.01–4.50

a GAE, gallic acid equivalent; QE, quercetin equivalent.

Proteins are the second most abundant component in BP. They constitute between 10 and 40% (w/w) of its dry weight. In addition to their nutritional contribution, they influence its taste value.^14^ The protein content may vary according to the type of plant, indicating a wide variation even between similar plant species from different geographical regions,^17,18^ and according to the collection method. The protein content in pollen collected by bees and hand-collected pollen is often high, probably related to the bees adding nectar to the pollen.^19^ Protein levels in bee pollen can also be affected by the harvest season. Pollen collected in spring showed the highest contents of crude protein and total amino acids as well as the highest levels of leucine, glutamic acid, valine, isoleucine, threonine, and glycine. On the other hand, the highest contents of phenylalanine, lysine, tryptophan, threonine, tyrosine, arginine, and cysteine were found in samples collected in winter. The highest contents of histidine, methionine, and serine were observed in BP collected in fall, while the highest levels of aspartic acid, proline, and alanine were identified in samples collected in summer.^20^ Protein levels in dehydrated BP can vary between 2.5 and 62 g/100 g (Table 1),^11^ mainly influenced by botanical origin,^11^ where the main protein fractions consist of albumins (35.4%), globulins (18.9%), glutelins (18.6%), prolamins (21.8%), and other proteins (including enzymes at 5.3%).^21^ BP also provides a total of 20 essential and non-essential amino acids.^10^ The amino acid profile and content of pollen do not focus only on nutritional value, because the level of certain amino acids could serve as an indicator of freshness, proper drying, and storage process. In addition, some of the acids have been proposed as possible botanical and geographical markers of bee pollen, mainly on the basis of the fact that botanical origin influences the amino acid profile more from a quantitative point of view than a qualitative point of view.^11^ Glutamic acid, proline, and aspartic acid have been described among the predominant amino acids in BP from various plant species and different geographical regions.^10^ However, other amino acids also stand out for their content, including leucine, lysine, threonine, histidine, tyrosine, and especially tryptophan (Table 1).^10,11^ The total essential amino acids in BP constitute between 12 and 45.02% of the total amino acid content,^11^ making it an important source of these macronutrients and suggesting their potential use as an innovative dietary supplement, especially for vegetarians and athletes.

Alongside carbohydrates and proteins, lipids are an important nutritional component of BP. Although the total lipid content in pollen from various plant species was previously reported in a range of 1–13% of dry weight,^22^ recent studies have revealed higher contents of up to 24.4% (Table 1).^17,23−25^ A total of nine lipid classes, including phosphatidylcholine (41 species), phosphatidylethanolamine (43 species), phosphatidylglycerol (9 species), phosphatidylserine (10 species), lysophosphatidylcholine (12 species), ceramide (8 species), diglyceride (27 species), triglyceride (137 species), and fatty acids (47 species), were reported in BP.^26^ In addition, a wide variety of up to 20 types of fatty acids (FAs) have been reported in BP: from C4 to C20, where ω-3 fatty acids are predominant.^10^ This content and variety are directly influenced by the predominant and secondary pollen botanical origins.^27^ Among the saturated acids, palmitic and myristic acids stand out for their content levels, followed by stearic and lauric acids in a lower concentration, while α-linolenic, γ-linolenic, and oleic acids constitute the most prominent unsaturated acids (Table 1). According to data collected in more than 100 BP studies from different geographical regions and botanical origin, saturated fatty acids represent between 4.29 and 71.47% of total lipids, while monounsaturated fatty acids range between 1.29 and 53.24%, and polyunsaturated fatty acids range between 4.33 and 75.7%. Meanwhile, ω-3 fatty acids ranged between 8.07 and 44.1%, and ω-6 fatty acids ranged between 1.77 and 38.25%.^9,10^

Despite the low number of reports available, BP is considered an important source of fat-soluble vitamins (0.1%), such as vitamin A in terms of β-carotene and tocopherols (vitamin E) (Figure S1 of the Supporting Information), as well as several water-soluble vitamins (0.3%) of vitamin B complex and vitamin C (Table 1).^11^ Dependent upon the season of the year, environmental conditions, geographical regions, and floral species, the content of certain vitamins in pollen varies.^28^ Among the fat-soluble vitamins, a wide range of α- and β-carotene contents has been reported in BP.^14^ Additionally, other carotenoids and their isoforms (γ-, ξ-, and ε-carotene) in *trans*- and *cis*-geometric shape (Figure S1 of the Supporting Information) have been reported in BP, standing out equally for their content levels,^29^ while four tocopherols (α-, β-, γ-, and δ-tocopherol) were also reported.^28^ With regard to the water-soluble vitamins, the main values reported correspond to vitamin B_3_ (nicotinamide and niacin) from Brazilian pollen^17^ as well as the vitamin C content, ranging from trace amounts to important contents, like 79.70 mg/100 g.^28^

BP also contains an important group of macro- and microelements, which vary according to different floral sources and geographical origins, where it has been suggested that soil type is the main cause of the variation.^30^ Up to 25 different compounds of this type have been reported in BP, where potassium (K), calcium (Ca), phosphorus (P), magnesium (Mg), sodium (Na), zinc (Zn), iron (Fe), manganese (Mn), copper (Cu), and selenium (Se) represent the most common elements reported worldwide (Table 1).^10,11^ The recommended daily amount of BP consumption for an adult is between 20 and 40 g.^9^ Nevertheless, considering the content of these elements, a moderate consumption of 25 g of BP could cover all or part of the recommended daily intake for most of the micro- and macroelements presented in Table 1, which highlights bee pollen as a relevant source of these elements. BP has also been proposed as a potential geographical marker.^31^ Therefore, the profile and mineral content of bee pollen could not only serve as food supplements but also as markers to identify its botanical origin and quality.

**Figure 1:**
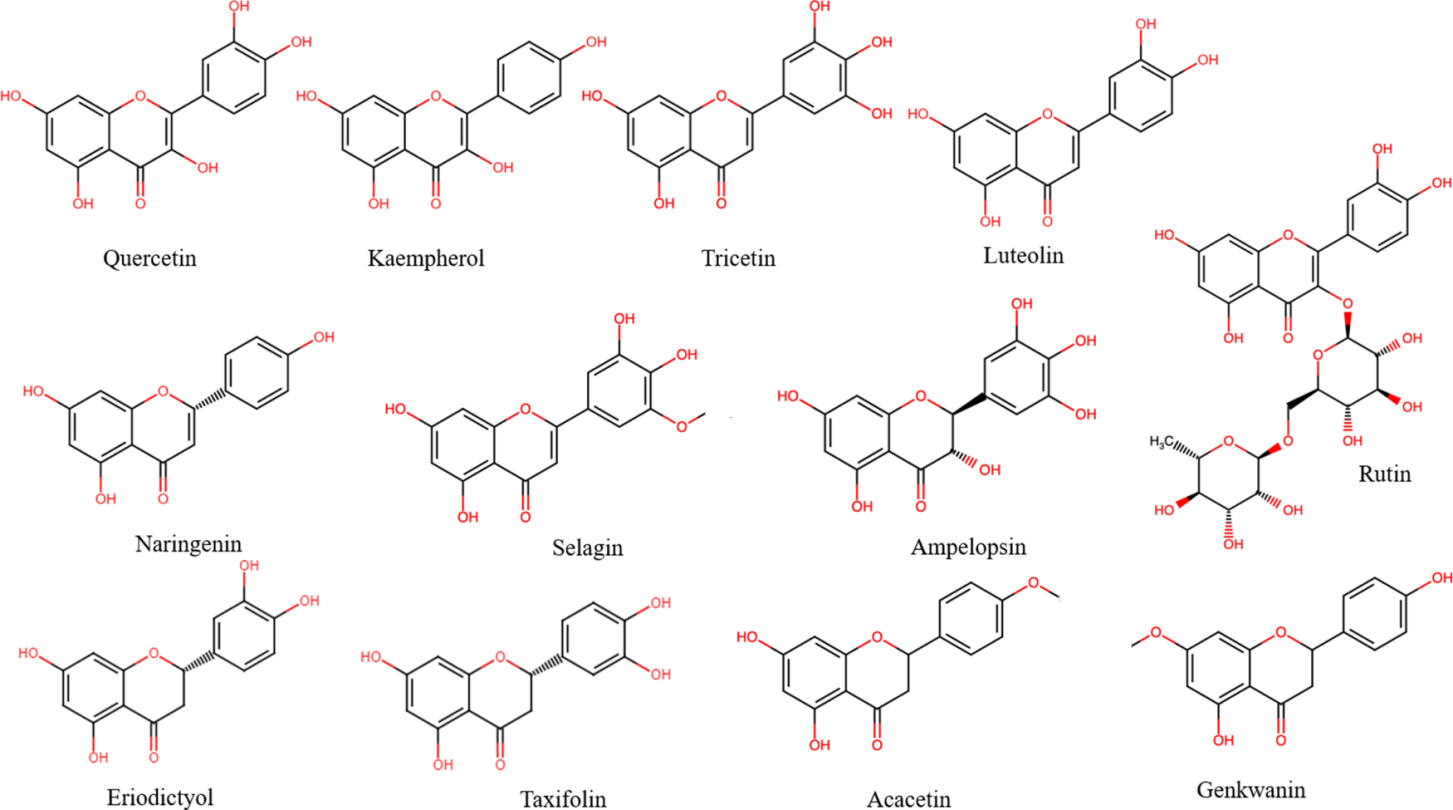
Chemical structures of the most representative flavonoids found in bee pollen.

**Figure 2:**
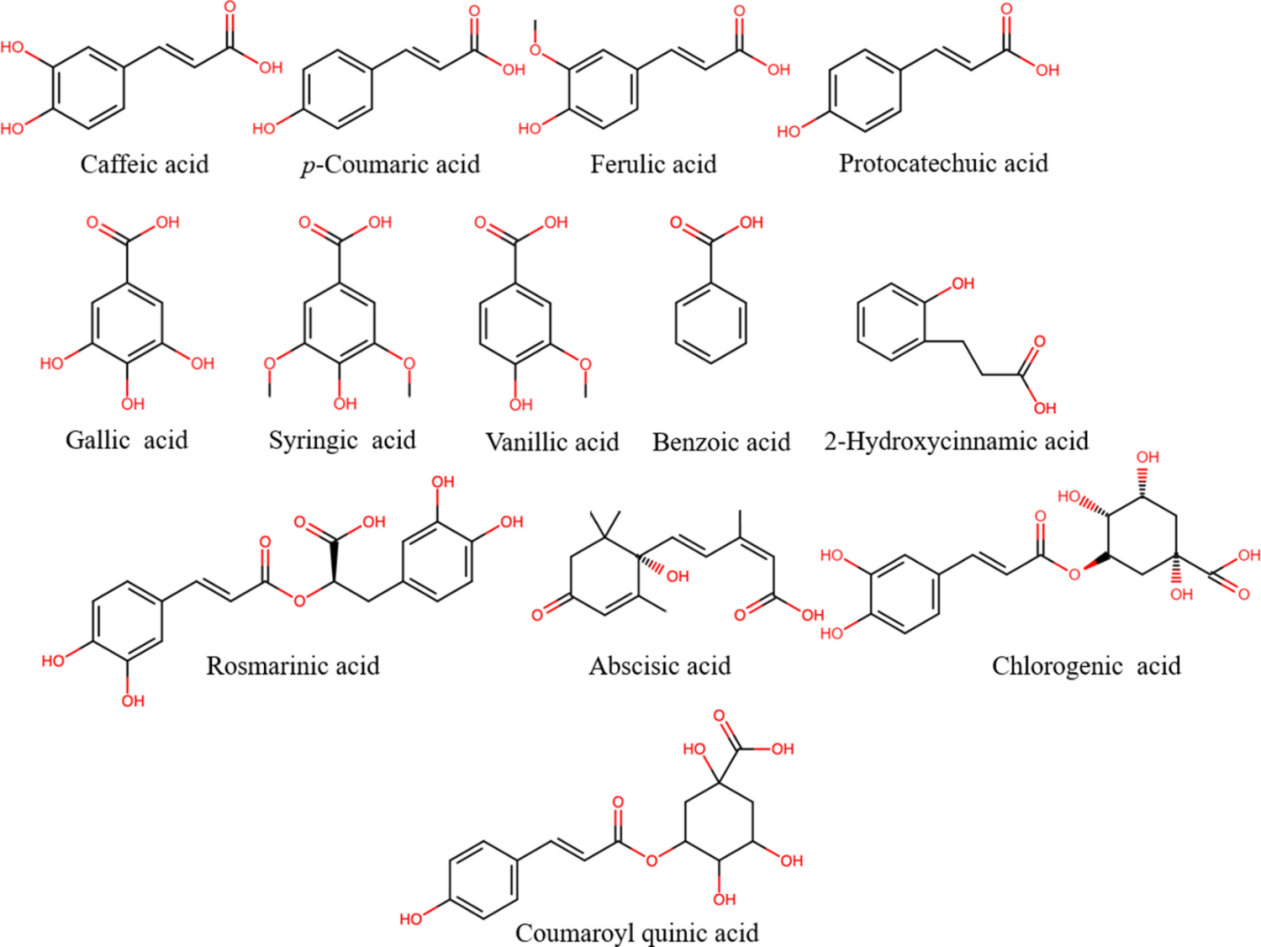
Chemical structures of the most representative phenolic acids found in bee pollen.

**Figure 3:**
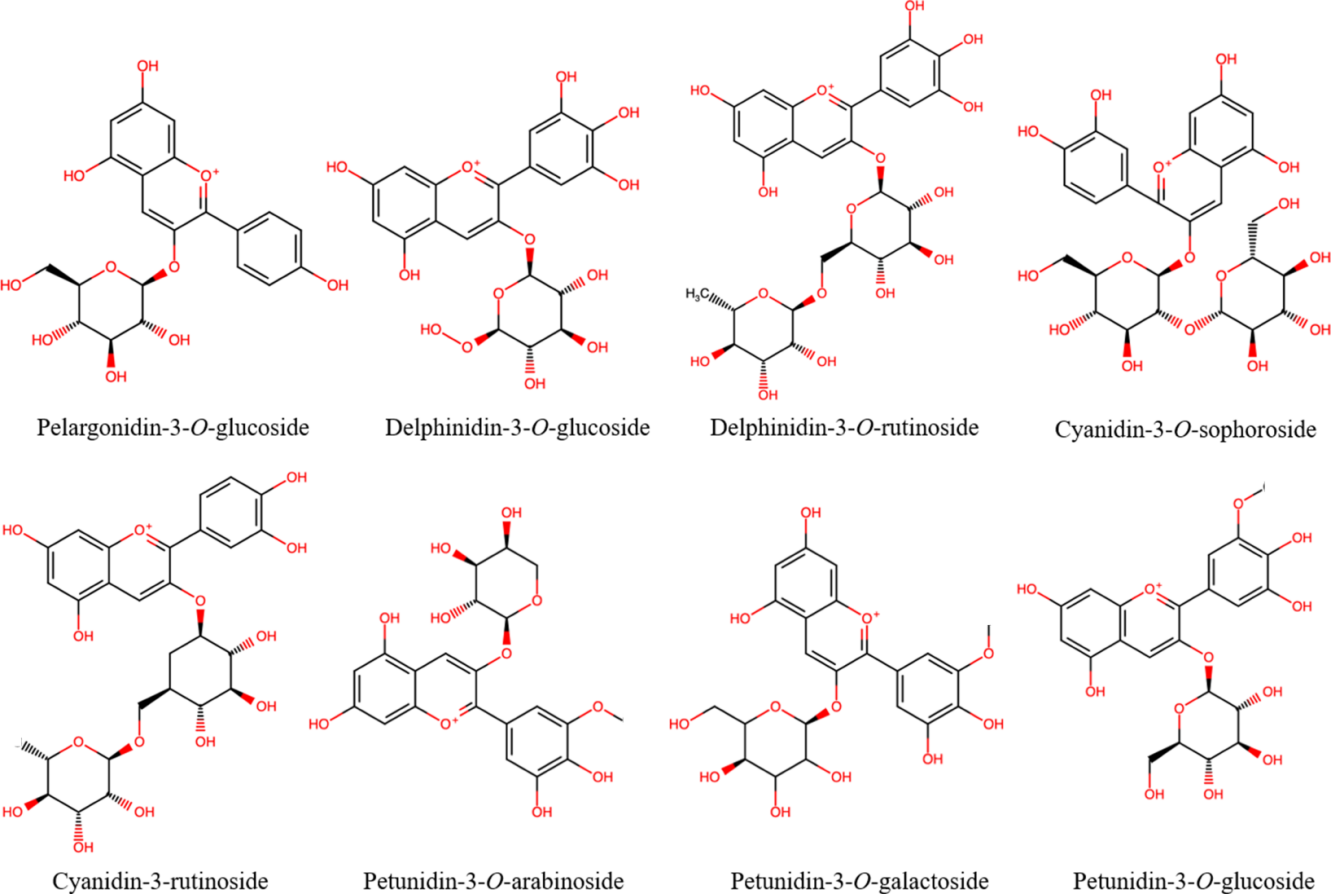
Chemical structures of the most representative anthocyanins found in bee pollen.

Given its floral origin, BP contains a wide group of polyphenolic compounds (Figures 1–3), mainly flavonoids and their glycosylated forms, along with phenolic acids and their derivates, which come from both the pollen itself and the nectar and honey that bees use to form the pollen granules. Phenolic compounds are responsible for the color of the pollen grain (yellow, brown, red, violet, etc.)^32^ as well as partly responsible for its most relevant biological activities, e.g., antioxidant activity.^33^ The phenolic acid and flavonoid contents in bee pollen show great variability (Table S1 of the Supporting Information)^9−11,13,14,34^ as a result of differing floral and geographical origins.^17^ Approximately 11 different phenolic acids and their derivatives or glycosylated forms (e.g., hydroxycinnamic acid glycosides) and hydroxycinnamic acid amide derivatives have been identified in BP from different geographical and floral origins,^9−11,35,36^ where ferulic acid (as a trace amount of 149 μg/g of pollen)^37−39^ and 2-hydroxycinnamic acid (43–180 μg/g of pollen)^39,40^ stood out for their content levels. BP also shows a broad flavonoid profile (Table S1 of the Supporting Information). About 19 different non-glycosylated flavonoids have been reported in bee pollen with a predominance of flavonols and flavone, followed by flavanones and, to a lesser extent, flavanonols and flavan-3-ols. Furthermore, a diverse group of glycosylated flavonoids, with a predominance of quercetin glycosides, has been identified, followed by isorhamnetin, myricetin, and kaempferol glycosides and, to a smaller degree, luteolin and apigenin.^9,10^ According to the concentration reports of these compounds, rutin (trace amount of 956 μg/g of pollen),^38−41^ and quercetin (trace amount of 530 μg/g of pollen)^37,38,40,41^ showed the highest concentrations among the non-glycosylated forms, while quercetin-3-*O*-β-d-glucosyl-(2→l)-β-glucoside (0.65–5108 μg/g of pollen) and kaempferol-3-*O*-β-d-glucosyl-(2→l)-β-d-glucoside and kaempferol-3,4′-di-*O*-β-d-glucoside (0.2–4243 μg/g of pollen) had significantly higher contents than the rest of the flavonoids.^42,43^

Along with its valuable nutritional contribution, BP also possesses potential therapeutic properties that are closely determined by its chemical composition. Given the polyphenol content of bee pollen, *in vitro* and *in vivo* models have demonstrated its outstanding antioxidant activity.^44−46^ BP has also been shown to have significant anti-inflammatory effects. *In vitro* studies have pointed out that this capacity is associated with the ability of BP compounds to suppress the production of pro-inflammatory cytokines, including cyclooxygenase 2, inducible nitric oxide synthase, interleukin (IL) 6, and tumor necrosis factor (TNF) α,^47^ as well as to downregulate inflammatory-related gene expression and block the activation of mitogen-activated protein kinase (MAPK) and nuclear factor κ-light-chain-enhancer of activated B cells (NF-κB) signaling pathways.^48^ Likewise, *in vivo* studies in rats demonstrated the anti-inflammatory effects of BP because of its capacity to regulate interferonγ (IFN-γ), noradrenaline, 5-hydroxytryptamine, dopamine, and caspase 3.^26^

The beneficial effect of BP consumption on the cardiovascular system has been linked to is hypolipidemic activity because it reduces the content of cholesterol, triacylglycerol, and total lipids.^49^ The regular intake of BP significantly decreases the level of lipids in blood correlated with the content of hormones, for instance, insulin, testosterone, and thyroxine.^49^ The daily supplementation of 40 g in patients with heart failure caused a reduction in the serum cholesterol level, blood viscosity, and fibrinogen and fibrin concentrations.^50^ Similarly, in humans supplemented with bee pollen, a decrease in the aggregation capacity of blood platelets and the formation of an atherosclerotic plaque has been confirmed as well as an increase in the activity of the fibrinolytic system, possibly related to the levels of ω-3 fatty acids, such as α-linolenic acid, that act as a prostaglandin 3 precursor and an inhibitor against platelet aggregation.^51^ Although it has been linked to allergic reactions, BP can be used to improve and even prevent certain allergic conditions, e.g., rhinitis and asthma. This effect is associated with its ability to obstruct the expression of β-hexosaminidase and reduce serum levels of immunoglobulin E (IgE) and immunoglobulin G1 (IgG1) as well as the inhibition of leukocyte migration to bronchoalveolar lavage.^52^ Moreover, researchers have found that it can also act to reduce degranulation in mast cells by inhibiting tyrosine protein phosphorylation. The lipid-soluble fraction of BP has also been proven to exert anti-allergic activity by hindering the binding of IgE to FcεRI in cutaneous cells.^53^ Although these results help to elucidate the possible anti-allergic activity of BP, the underlying mechanisms between anti-allergic activity and immune responses should be studied in greater depth.

BP has also been shown to possess relevant antimicrobial activities on several Gram-positive and Gram-negative pathogenic strains (*Escherichia coli*, *Staphylococcus epidermidis*, *Staphylococcus aureus*, *Staphylococcus pyogenes*, *Salmonella enteritidis*, *Listeria monocytogenes*, and *Pseudomonas aeruginosa*)^54^ as well as against different strains of fungi and yeasts (*Aspergillus fumigatus*, *Aspergillus flavus*, *Aspergillus niger*, *Candida albicans*, *Candida glabrata*, *Candida krusei*, *Candida parapsilosis*, *Candida tropicalis*, *Geotrichum candidum*, *Fusarium culmorum*, *Penicillium verrucosum*, and *Rhodotorula mucilaginosa*).^55,56^ Despite this broad spectrum of antimicrobial activity, BP can be a suitable substrate for the growth of mycotoxins, such as aflatoxins, if no adequate drying is done by the beekeeper, so that the strict control of toxins should be considered when considering the quality standards of BP.^57^

## 3 Bee Bread

One of the least known and investigated bee products is bee bread (BB). Honeybees cop with their nutritional needs by collecting water, nectar, and pollen. Carbohydrates are mainly provided by nectar but also by pollen. However, the rest of the nutrients, including lipids, proteins, minerals, and vitamins, comes almost exclusively from pollen. BB is produced when bees collect the pollen and mix it with saliva and honey, and this mixture is packed into the cells of the honeycomb. Under the action of temperature (35–36 °C), moisture, activity of different enzymes from glandular secretions, and microorganisms (including bacteria and yeasts), after 2 weeks, BB is formed. During winter and early spring, BB together with honey is the only source of food for the bee colony. Moreover, BB is used to produce royal jelly by young bees and worker bees to fed larvae.^9,14^

The composition of BB is mediated by its source materials as well as the physical–chemical and microbiological processes that take place during its production.^9,14^ Thus, BB is produced with considerable variations in its centesimal composition (in both macro- and micronutrients and even non-nutritional bioactive compounds) depending upon the geographical and botanical origin of the pollen as well as the microbiota present in the cell. Other factors affecting BB composition are the climatic conditions, soil type, beekeeper activities, or storage treatments in commercial production.^58^ Overall, water in BB (Table 2) represents up to 30% of fresh weight. Concerning carbohydrates (Table 2), analyzed studies reported proportions ranging from 13 to 72% of dry weight. Among the observed carbohydrates, different sugars can be found (Table 2), mainly fructose, glucose, sucrose, trehalose, and maltose but also fructofuranose α and β, α glucopyranose, mannitol, β-d-glucopyranose, melezitose, and raffinose. In the process of making BB, the carbohydrate content is increased as a result of the addition of nectar and honey with the fresh pollen. Moreover, the sugar content varies because the action of bee salivary glands. Thus, the action of α- and β-amylase and α-glucosidase results in the breakdown of polysaccharides into simple sugars. Glucose and fructose are required by bacteria to ferment bee pollen into BB that is digested into ethanol, lactic acid, and carbon dioxide by lactic acid bacteria; glucose and fructose can also be transformed into mannitol after mixing with the bee salivary enzymes. All of these processes and probably others are responsible for the final sugar content available in BB.^9,14,58^

**Table 2:** Bee Bread (BB) Composition from Different Geographical and Botanical Origins^a^ ^58−60,62−65,67,68^

proximate	content (min–max)		
moisture (%)	5.91–30.12		
protein (%)	17.11–30.34		
ashes (%)	1.93–3.42		
lipids (%)	1.95–11.55		
carbohydrates (%)	13.02–72.23		
sugars (g/100 g)			
fructose	0.994–19.73		
glucose	8.82–12.40		
maltose	0.945–1.244		
sucrose	0.845–1.492		
trehalose	0.544–0.921		
melezitose	0.97–1.15		
raffinose	0.96–1.24		
polyphenols			
total phenolic content (mg of GAE/g)	2.53–13.75		
total flavonoid content (mg of QE/g)	1.94–4.51		
TEAC (μmol of Trolox/g)	46.13–76.38		
FRAP (μmol of Trolox/g)	35.03–70.17		
amino acids (g/100 g)			
essential amino acids		non-essential amino acids	
arginine	0.11–2.76	alanine	0.5–1.7
histidine	0.82–1.21	aspartic acid	1.4–5.2
isoleucine	0.45–0.94	glutamic acid	0.2–1.7
leucine	0.16–2.13	glycine	0.4–1.4
lysine	0.53–0.89	proline	1.5–22.2
methionine	0.14–0.53	serine	0.6–2.2
phenylalanine	1.89–3.32	tyrosine	0.6–1.6
threonine	0.18–2.01	asparagine	2.47–5.89
tryptophan	0.14–1.42	glutamine	0.04–0.34
valine	0.17–1.20		
fatty acids (g/100 g)			
C4:0 butyric acid	0.75–1.29	C20:1 eicosenoic acid	0.28–6.28
C6:0 caproic acid	0.08–0.35	C21:1 heneicosenoic acid	0.39–0.43
C8:0 caprylic acid	0.02–0.34	C20:2 eicosadienoic acid	0.08–1.73
C10:0 capric acid	0.02–0.19	C20:3 dihomo-γ-linolenic acid	0.02–2.10
C11:0 undecanoic acid	0.07–0.90	C20:3 eicosatrienoic acid	0.47–0.52
C12:0 lauric acid	0.05–6.15	C20:4 arachidonic acid	0.02–25.12
C14:0 myristic acid	0.21–1.36	C22:0 behenic acid	0.08–2.60
C15:0 pentadecanoic acid	0.14–0.53	C22:1 erucic acid	0.11–5.43
C16:0 palmitic acid	10.21–38.69	C22:6 docosahexaenoic acid	0.04–0.29
C16:1 palmitoleic acid	0.05–1.14	C23:0 tricosanoic acid	0.58–5.61
C17:0 heptadecanoic acid	0.20–0.91	C20:5 eicosapentaenoic acid	0.05–0.64
C17:1 heptadecenoic acid	0.11–0.49	C24:0 lignoceric acid	0.03–1.65
C18:0 stearic acid	1.31–6.40	C24:1 nervonic acid	0.08–1.21
C18:1 *cis*-oleic acid	3.90–21.25	SFA	23.15–28.68
C18:2 *trans*-linoelaidic acid	0.03–0.22	UFA	71.03–77.32
C18:2 *cis*-linoleic acid	6.26–36.96	MUFA	31.11–72.15
C18:3 α-linolenic acid	0.17–40.02	PUFA	45.54–64.70
C20:0 arachidic acid	0.61–4.7		
minerals (mg/kg)			
macrominerals		microminerals	
potassium (K)	3380.21–7551.54	zinc (Zn)	44.91–331.03
calcium (Ca)	1455.21–1980.21	iron (Fe)	119.52–273.61
phosphorus (P)	2510–6577.11	manganese (Mn)	37.27–890.20
magnesium (Mg)	610.21–2000.20	copper (Cu)	6.80–7.20
sodium (Na)	115.70–155.51	selenium (Se)	0.18–0.90
vitamins (mg/100 g)			
fat soluble		water soluble	
tocopherols (vitamin E)	10.01–11.22	vitamin C	10.87–11.52

a GAE, gallic acid equivalent; QE, quercetin equivalent; FRAP, ferric-ion-reducing antioxidant power; MUFA, monounsaturated fatty acid; PUFA, polyunsaturated fatty acid; SFA, saturated fatty acid; UFA, unsaturated fatty acid; and TEAC, Trolox equivalent antioxidant capacity.

Proteins represent between 17 and 30% of dry weight according to the reviewed literature.^9^ According to Mohammad et al.,^59^ protein use is higher in BB than in bee pollen (BP), while on other hand, Zuluaga and co-workers^60^ described that the content of protein in BB can be similar to BP because the biochemical process induced by bees is aimed at degrading the outer layer of the pollen grain, without any damage of the inner content. However, because of the addition of sugar by the bee, BB protein can be unpredictable.^61^ With regard to the amino acidic profile, the content is higher compared to BP, which has been suggested to happen because of degradation of pollen protein, leading to more peptides and amino acids.^9,59^ The microbial presence might be responsible for both the increase of amino acids through proteolysis and decrease of amino acids by its use as an energy source. It has been described as an important number of amino acids present in different BB samples with a very variable content.^62^ A summary with the observed range can be shown in Table 2.

The lipid content in BB may vary from 1.1 to 11.55% of dry weight.^63^ Particular attention has been focused on the fatty acid profile of BB (Table 2). Up to 37 fatty acids have been described in BB.^64^ Unsaturated fatty acids (UFAs) predominate over saturated fatty acids (SFAs), with up to 77.32% of total fatty acids with respect to 28.68%, respectively. Among UFAs, there is a good correlation between monounsaturated fatty acids (MUFAs) and polyunsaturated fatty acids (PUFAs), with a maximum of 72.15% and a 64.70%, respectively. The presence of some essential PUFAs in BB is important, which is very relevant from the point of view of human health. From the point of view of bees, it has been described that some fatty acids, like oleic and palmitic acids, are important for nutrition. On other hand, myristic, linoleic, and linolenic acids are relevant because they can inhibit the growth of spore-forming bacteria.^63^

BB is also a moderate source of organic acids, such as gluconic, formic, acetic, propionic, and butyric acids,^65^ and even probiotics, including lactic acid.^66^

Ashes represent between 1.93 and 3.42% of dry weight.^60,64,65^ An important fraction of ashes is formed by minerals (Table 2). Considering the studies in which the most accurate technology has been used to analyze minerals, i.e., inductively coupled plasma mass spectrometry, the most abundant mineral reported for BB is K, followed by P, Ca, and Mg.^59,63^ As for BP, the principal origin of minerals is pollen and soil, but in BB, also nectar is a relevant source of these chemical elements. Concerning vitamins, it is expected that, because BB is derived from pollen, which is rich in vitamins, BB also has them. However, the literature is scarce on this matter. In fact, despite the scientific literature when describing BB composition, no mention is made on its content in various fat- and water-soluble vitamins; after an exhaustive search for vitamin values, only levels of tocopherol and vitamin C have been documented in recent literature (Table 2).

Because one of its main components is pollen, the presence of phenolic compounds is expected in BB. Table 2 shows the total contents of phenols and total flavonoids according to the literature. Concerning individual compounds, research on the subject indicates the presence of numerous phenolic molecules in BB.^14,58,62,63,65^ Urcan et al.^67^ found that the phenolic profile of BB is similar to that of BP (Table 2), despite the transformations that take place in pollen during the manufacture of BB. Rutin is the main component observed, and quercetin is also present in an important concentration.^62^ Transformations during BB production as a result of, for example, bacterial action could modify the relative proportions of various compounds between BP and BB. Thus, Bayran et al.^62^ have observed some compounds to be higher in BP (caffeic acid, rutin, ethyl gallate, *trans*-ferulic acid, and myricetin) and in other cases higher in BB (protocatechuic acid, *p*-coumaric acid, quercetin, 2,5-dihydroxybenzoic acid, kaempferol, gallic acid, chlorogenic acid, salicylic acid, luteolin, and isorhamnetin).

In the last years, numerous investigations have been conducted to study its biomedical potential that is strictly correlated with the source pollens and, consequently, the nutrient and non-nutrient compounds present in BB.^58^ Because of the presence of phenolic compounds and other ingredients, the antioxidant capacity has often been analyzed. Table 2 presents typical values for Trolox equivalent antioxidant capacity (TEAC) and ferric-ion-reducing antioxidant power (FRAP) from the literature. It has been described that BB has a similar or higher antioxidant capacity than honey or propolis.^63^ The antimicrobial effect of BB has been extensively investigated. Bakour et al.^63^ found that different bacterial strains and fungi were sensitive to a hydromethanolic BB extract. It has been described that BB extracts are more effective against Gram-positive bacteria compared to Gram-negative bacteria.^68^ Antitumoral activity has also been assayed *in vitro* in some studies.^69^ Additionally, some investigations have been conducted regarding anti-inflammatory and immunomodulatory effects, antihypertensive activity, hypolipidemic effect, and hepatoprotective actions.^58^ More standardized and systematic experimental research is needed to know in depth the biomedical properties of BB beyond its antioxidant and antimicrobial capacity, aspects on which more information is available to date.

## 4 Royal Jelly

Royal jelly (RJ) is a gelatinous and creamy secretion produced by the hypopharyngeal and mandibular glands of the young honeybee (*A. mellifera* L.) workers, named nurses.^70^ It is usually used to feed the larvae of bee workers until the third day of their life (after this period, they are nourished with a mix of honey, RJ, and pollen), while it represents the unique food for the queen bee for her entire life.^71−73^ The consistency of RJ is not always homogeneous because some undissolved pollen grains of different sizes may persist; it is moderately acidic, with a pH ranging from 3.1 to 3.9 and a density of 1.1 g/mL; its color varies from white to yellow; and its odor and taste are lightly pungent.^74−76^

With regard to the chemical and nutritional composition, RJ is essentially made up of water (60–70%), carbohydrates, proteins and free amino acids, lipids, minerals, vitamins, and polyphenols.^77^ Sugar contents range between 7 and 18%, with glucose and fructose the most representative sugars, accounting for about 90% of the total sugars found in RJ; other sugars that may be present in smaller quantities are ribose, sucrose, trehalose, maltose, galactose, erlose, and melibiose.^78,79^

Protein contents vary between 9 and 18%, of which 80% is represented by the so-called major royal jelly proteins (MRJPs). These proteins are water-soluble and comprise nine members MRJP1–MRJP9, with molecular masses of 49–87 kDa.^80,81^ From the structural point of view, MRJPs have repetitive pentapeptide zones enriched with nitrogen-rich amino acids or repetitive tripeptide zones enriched with methionine (i.e., MRJP5);^81^ they exert essential physiological effects in the development of the queen bee.^82^ Beside MRJPs, other proteins present in small amounts in RJ are aspimin, jelleines, royalisin,^78^ and small peptides, such as dipeptides (i.e., Lys-Tyr, Arg-Tyr, Ala-Leu, Phe-Lys, Ile-Arg, Lys-Leu, Arg-Tyr, Tyr-Tyr, and Tyr-Tyr) with high antioxidative and antibacterial activities.^78,83,84^ RJ is also rich in free amino acids, with glycine, lysine, glutamic acid, and proline being the most abundant, followed by alanine, leucine, isoleucine, arginine, phenylalanine, aspartic acid, threonine, serine, methionine, valine, and tyrosine.^71,80,85^

Fatty acids, waxes, phenols, steroids, and phospholipids constitute the lipid fraction of RJ (3–8%). Specifically, fatty acids contain 8–12 carbons and are typically either dicarboxylic or hydroxyl fatty acids. They include *trans*-10-hydroxy-2-decenoic acid, gluconic acid, and 10-hydroxydecanoic acid that are the main fatty acids in RJ from the quantitative point of view, followed by 9-hydroxydecanoic, 7- and 8-hydroxyoctanoic, 9-hydroxy-2-decenoic, 3-hydroxydecanoic, 3,10-dihydroxydecanoic, and 10-hydroxydecanoic acids; 2-decene-1,10-dioic and 2-octene-1,8-dioic acids have been also reported in RJ, together with the mono- and diesters of 10-hydroxy-2-*trans*-decenoic acid and hydroxyl-2-*trans*-decenoic acid 10-phosphate.^72,74,86−88^ Finally, 24-methylenecholesterol and Δ^5^-avenasterol are the main sterols found in RJ.^80^

With regard to micronutrients, the most abundant minerals in RJ include Ca, Cu, Fe, K, Mg, Mn, Na, and Zn, followed, in trace amounts, by aluminum, antimony, barium, bismuth, cadmium, cobalt, chrome, lead, thallium, and vanadium, among others,^75,87^ while pantothenic acid is the most abundant vitamin present in RJ, followed in trace amounts by ascorbic acid, biotin, folic acid, niacin, pyridoxal, riboflavin, and thiamine.^70,89^ RJ may also contain nucleotides, as both free bases, including adenosine, cytidine guanosine, uridine, and phosphates, comprising adenosine mono-, di-, and triphosphate.^78^

The composition of bioactive compounds in RJ is strictly correlated with the regional and seasonal conditions. The phenolic content varies between 3.1 and 15.4 mg of gallic acid equivalent (GAE)/g and comprises mainly flavonoids, phenolic acids, organic acids, and their esters (Table S2 of the Supporting Information).^78,90−92^ The main volatile compounds found in RJ are esters, aldehydes, ketones, acids, and alcohols (Table S2 of the Supporting Information), but their presence and concentrations strictly depend upon several factors, such as honeybee species, geographical area, harvest time, storage type, and processing system, making the comparison among different RJ samples difficult to do.^90,93,94^

The presence and amount of the different macro- and micronutrients and bioactive compounds determine the biological properties of RJ. For example, several RJ samples from different geographical zones have been found to exert important (i) antibacterial activities against both Gram-positive (*Staphylococcus aureus*, *Staphylococcus epidermidis*, *Micrococcus luteus*, *Listeria monocytogenes*, and *Streptomyces griseus*) and Gram-negative (*Proteus vulgaris*, *Escherichia coli*, *Pseudomonas aeruginosa*, *Enterobacter cloacae*, and *Klebsiella pneumoniae*) bacteria, (ii) anti-inflammatory effects in different *in vitro* and *in vivo* models, through the decrease of pro-inflammatory cytokine secretion and the modulation of different molecular pathways, such as TNF-κB, IL-1β, IL-18, and MAPK, and (iii) antioxidant properties in some *in vitro* and *in vivo* studies, with the ability of RJ to act as radical scavengers and affect protein expression in a dose-dependent manner.^95^ Other biological activities exerted by RJ include the immunomodulatory effects,^95−97^ vasodilative, hypotensive, and antihypercholesterolemic activities,^95,98−102^ and antitumor properties.^103−108^ In particular, 10-hydroxy-2-decenoic acid, the main lipid component of RJ, has been associated with several biological effects, including estrogenic, antimicrobial, anti-inflammatory, antitumor, and immunomodulatory activities as well as the increase of the lifespan of *Caenorhabditis elegans* through dietary restriction and target of rapamycin signaling, inhibition of the vascular endothelial growth factor, activation of TRPA1 and TRPV1 receptors, induction of neurogenesis, suppression of skin pigmentation, and protective effect against ultraviolet B in the human skin.^109^

## 5 Propolis

Propolis, also named bee glue, is a sticky resinous substance that honeybees (*Apis* spp.) collect from living plants. It is a complex mixture, made essentially by plant resins (50%), waxes (30%), aromatic and essential oils (10%), pollens (5%), and other organic compounds (5%);^110^ according to the botanical source, its color may range from green to reddish and brown.^111^ Propolis is usually used by honeybees to maintain a stable temperature and moisture inside the hive as well as to smooth walls and seal cracks. Moreover, it is also used by bees to mummify dead invader insects that are too heavy to be removed from the colony.^112^

Before commercialization, it must be purified through the use of different methods, including, for example, shaking, soaking, Soxhlet extraction or reflux, and different solvents, such as absolute ethanol or aqueous ethanol at 70–95%, pure water, hexane, acetone, methanol, chloroform, etc., to remove the undesired material; the type of extraction and the solvents used strongly affect the yields of bioactive compounds that remain in propolis.^110^

With regard to the chemical and nutritional composition of propolis, the main group of compounds is represented by phenolic components, mainly flavonoids, phenolic acids, lignans, stilbenes, and coumarins (Table S3 of the Supporting Information); other minor components are beeswax, lipid–wax substances, terpenes, resins, balms, and sugars as well as mono- (glucose and fructose) and disaccharides (sucrose), proteins, amino acids, minerals (Ca, Cu, K, Mg, Mn, Na, and Zn), and vitamins (B_1_, B_2_, B_6_, C, and E) that account for a negligible part of propolis composition.^110,113−117^

**Figure 4:**
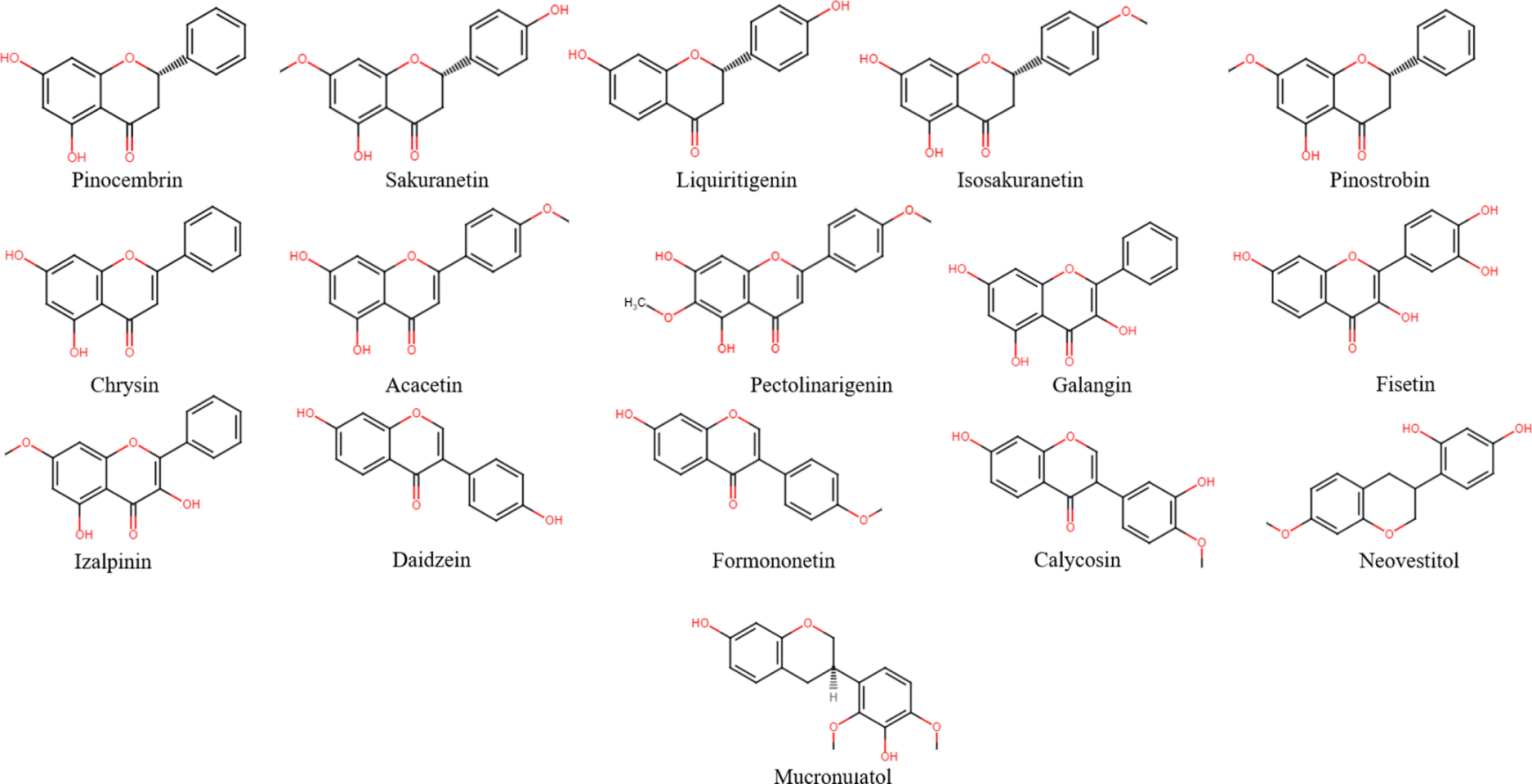
Chemical structures of the most representative flavonoids found in propolis.

The phenolic profile of propolis is strongly influenced by the geographical area and the climatic conditions where plants grow; for example, in Europe, North America, and Asia (temperate zone), phenolic patterns are characterized by high levels of flavonoids (mainly flavones and flavanones) and low levels of phenolic acids, while in tropical areas, propolis has more complex composition, with prenylated flavonoids, prenylated *p*-coumaric acids, and lignans, among others.^118^ The total phenolic content usually ranges from 127 to 142 mg of GAE/g, while the total flavonoid concentration varies between 33 and 53 mg of quercetin equivalents/g.^119^ The total flavonoid content represents an index of quality of raw propolis: if it is less than 11%, crude propolis is considered of low quality; between 11–14%, acceptable; between 14–17%, good; and >17%, high quality.^120^ Until now, more than 150 flavonoids have been identified in propolis, including flavones, flavanones, flavanonols, flavonols, isoflavones, isodihydroflavone, flavan, and isoflavan, according to the plant source.^110,118^ Generally, these compounds are in the form of aglycone, because β-glucosidase secreted by bees usually removes sugar residues from most of the flavonoids present in the plants,^118^ even if it could be ineffective in hydrolyzing some glycosides, such as *C*-glycosides or β-diglycosides; this could explain, for example, the presence of quercetin 3-*O*-rutinoside, isorhamnetin 3-*O*-rutinoside, kaempferol rutinoside, flavone *C*-glycosides, naringenin, and luteolin glucosides in propolis from different countries.^118^ The most representative flavonoids found in propolis are illustrated in Figure 4.

Hydroxybenzoic and hydroxycinnamic acids are the main phenolic acids of propolis collected in different parts of the world, from Brazil to Australia: the first comprises gallic, gentisic, protocatechuic, salicylic, and vanillic acids, and the latter includes *p*-coumaric, caffeic, and ferulic acids. Moreover, besides the free forms, propolis can also contain the conjugated forms of phenolic acids, such as cinnamyl, benzyl, phenylethyl, and methyl butenyl esters. For example, in propolis collected in the temperate zone, caffeic acid phenethyl ester is the main component, while hydroxycinnamic acid derivatives are common in Australian propolis; in addition, according to the plant source, in Brazilian samples, chlorogenic acids and prenylated phenylpropanoids, such as artepillin C or chromene, were also shown to be very common.^118^

Lignans are present in few samples collected especially in tropical zones, mainly Brazil,^121^ and the Canary Islands,^122^ especially as syringaresinol and pinoresinol, while stilbenes have been reported in samples from Kenya, the Solomon Islands, and Australian Kangaroo Island, specifically in the form of geranylstilbenes (scheweinfurthin A and B), 5-farnesyl-3′-hydroxyresveratrol, pterostilbene, pinosylvin, O-prenylated tetrahydroxystilbenes, and C-prenylated tetrahydroxystilbenes;^123^ some coumarins have also been identified in a few samples^124^ (Table S3 of the Supporting Information).

Finally, another class of chemical constituents present in propolis is represented by volatiles.^125^ Even if they are minority compounds, they are important because they confer to propolis, from different origins, the typical aroma. The type and amount of volatiles in propolis depend upon several factors, including the type of bee, the geographical area, the plant, and also the methods used for their extraction and analysis. The main volatiles found in propolis are present in Table S3 of the Supporting Information.

Even if the use of propolis in medicine dates back to ancient times, only recently, its use for preventive and therapeutic purposes has been re-evaluated in a more scientific way. Several studies have indeed demonstrated the multiple biological properties of propolis, including the antioxidant, anti-inflammatory, immune-stimulating, antimicrobial, and even anticancer activities, highlighting the strong relationship between these effects and the chemical composition of propolis. For example, the antioxidant capacity can be ascribed to the presence of phenolic compounds of propolis that has been found to decrease oxidative stress in different *in vitro* and *in vivo* experimental models, including neuronal cell cultures and fibroblasts stressed with hydrogen peroxide or in mice treated with 1,1-diphenyl-2-picrylhydrazyl (DPPH).^126^

With regard to anti-inflammatory and immune-modulatory effects, propolis seems to play a key role, by inhibiting, for example, the release of TNF-α and IFN-γ and the degradation of tryptophan in stimulated peripheral blood mononuclear cells,^127^ upregulating the expression of TLR-4 and CD80 expression and downregulating the production of TNF-α and IL-10 in human monocytes, increasing the levels of anti-inflammatory cytokines IL-4 and IL-10 in mice blood,^128^ or increasing the expression of TLR-2 and TLR-4 and the production of IL-1β and IL-6 in the spleen cells of mice.^129,130^

The most studied and recognized property of propolis is the antimicrobial activity that mainly depends upon the synergistic effects of its several antimicrobial components (especially flavonoids and phenolic acids) rather than individual components. Specifically, propolis is active against several aerobic and anaerobic Gram-positive (*Bacillus cereus*, *Enterococcus faecalis*, *Micrococcus luteus*, *Nocardia asteroides*, *Staphylococcus aureus*, *Staphylococcus epidermidis*, *Staphylococcus haemolyticus*, *Streptococcus faecalis*, *Streptococcus pneumioniae*, *Streptococcus pyogenes*, *Streptococcus haemolyticus*, *Streptococcus mutans*, *Actinomyces naeslundii*, *Lactobacillus acidophilus*, and *Peptostreptococcus micros*) and Gram-negative (*Aeromonas hydrophila*, *Brucella abortus*, *Corynebacterium pseudotuberculosis*, *Escherichia coli*, *Helicobacter pylori*, *Klebsiella pneumoniae*, *Salmonella enteritidis*, *Salmonella typhi*, *Salmonella* Typhimurium, *Pseudomonas aeruginosa*, *Proteus mirabilis*, *Proteus vulgaris*, *Shigella dysenteriae*, *Porphyromonas gingivalis*, *Fusobacterium nucleatum*, and *Prevotella oralis*) bacteria, among others; it is also effective against different viruses, including adenovirus, influenza viruses, herpes simplex virus, poliovirus, rotavirus, coronavirus, and vesicular stomatitis virus, protozoa, such as *Plasmodium falciparum*, *Plasmodium malariae*, *Trichomonas vaginalis*, *Trypanosoma brucei*, *Trypanosoma evansi*, *Trypanosoma cruzi*, *Giardia duodenalis*, *Giardia lambia*, and *Entamoeba histolytica*, helminths, comprising *Taenia saginata*, *Fasciola gigantica*, and *Toxocara vitulorum*, and fungi, especially different strains of *Candida*, as well as *Microsporum gypseum*, *Trichophyton rubrum*, and *Trichophyton mentagrophytes*.^126^

Thanks to its antioxidant, anti-inflammatory, and antimicrobial activities, propolis is effective in (i) wound treatment, including surgical wounds, infected wounds, gastric ulcers, and diabetic ulcers, (ii) diabetic conditions, by decreasing the levels of blood glucose, glycated hemoglobin, and oxidative stress, enhancing the antioxidant systems, and improving the lipid profile in animal models of diabetes and atherosclerosis, and (iii) proliferative disorders, including colon, breast, prostate, brain, lung, and cervical cancer, both *in vitro* and in animal studies.^126^

## 6 Beeswax

Beeswax (BW) is the product of the wax glands of bees that use it to build their combs.^131^ BW is obtained by melting the combs of the hives after the honey has been removed from them. The combs are melted using steam or boiling water. The wax obtained after pressure filtration is called yellow beeswax. During the process, activated carbon or diatomaceous earth can be used to remove certain impurities. A more aggressive procedure on yellow beeswax, such as bleaching the natural pigments by exposure to sunlight, activated carbon, diatomaceous earth, and other earth or peroxides, leads to the production of white beeswax.^131^ According to the European Food Safety Authority (EFSA), “beeswax is a complex mixture of saturated and unsaturated linear and complex monoesters, hydrocarbons, free fatty acids, free fatty alcohols and other minor substances produced by the worker honeybee”.^132^ In 1980, Tulloch reported that more than 300 individual components are present in BW.^133^ Additionally, around 50 aroma components have been identified in this wax.^134^ For the same species, BW composition is quite stable, with small changes in proportions. More relevant differences are present between species.^131^ Main components are shown in Table 3, together with the detailed composition in some groups of molecules, including fatty acid monoesters, which represent, from a quantitative point of view, the most important compounds in BW. Also, the content in aliphatic hydrocarbons, total fatty acids, total alcohols, and chemical elements can be found in Table 3.

**Table 3:** Beeswax Composition from Different Geographical and Botanical Origins^50,132,133,138,139^

main component (%)	content (min–max)		
monoesters	27–40		
hydroxymonoesters	9–23		
diesters	7–16		
hydroxydiesters	3–9		
hydrocarbons	11–28		
free fatty acids	1–18		
free fatty alcohols	0–0.3		
others	4–8		
monoesters (%)			
C40	10.13–10.27	C46:1	0.02–0.22
C42	5.80–5.99	C48	8.16–8.31
C42:1	0.22–0.25	C48:1	0.31–0.35
C44	6.27–6.60	C50	0.56–0.83
C44:1	0.19–0.23	C50:1	0.54–0.92
C46	13.75–16.49		
aliphatic hydrocarbons (%)			
H19	0.14–0.22	H33:1	1.77–4.31
H20	0.05–0.39	H33	0.39–0.74
H21	0.10–0.43	H34	0.11–0.12
H22	0.03–0.15	H35:1	0.11–0.23
H23	0.71–3.08	H35	0.42–0.46
H24	0.07–0.19	H36	0.00–0.16
H25	1.79–5.51	H37	0.05–0.11
H26	0.20–0.39	H38	0.00–0.03
H27	3.17–15.96	H39	0.02–0.09
H28	0.18–0.41	H40	0.09–0.11
H29	1.99–9.53	H41	0.01–0.09
H30	0.13–0.32	H42	0.02–0.04
H31:1	0.82–2.79	H43	0.01–0.06
H31	2.27–7.47	H44	0.00–0.01
H32	0.09–0.37		
total fatty acids			
C14	0.09–0.11	C24	4.21–5.11
C16	16.28–19.22	C26	1.50–1.95
C18:2	0.03–0.11	C28	1.43–2.11
C18:1	2.13–2.34	C30	1.55–1.98
C18	0.51–0.67	C32	1.50–1.85
C20	0.03–0.11	C34	1.43–1.90
C22	0.43–0.65	C36	0.24–0.65
total alcohols (%)			
C22-OH	0.05–0.09	C32:1-OH	0.25–0.83
C24-OH	5.45–7.12	C32-OH	8.25–10.87
C26-OH	4.45–6.25	C34:1-OH	0.08–0.41
C28-OH	4.85–6.12	C34-OH	1.12–1.87
C30-OH	9.18–11.25		
chemical elements (mg/kg)			
Mg	17.02–27.50	Co	0.00–0.01
Al	7.55–14.31	Zn	9.70–11.42
Si	0.00–3.30	As	0.01–0.02
P	45.08–50.62	Se	0.00–0.03
K	78.65–99.56	Y	0.01–0.02
Ca	76.05–302.43	Cd	0.00–0.04
V	0.01–0.03	Au	0.00–3.51
Mn	0.26–0.84	Hg	0.20–0.72
Fe	16.90–24.76	Pb	0.00–4.48

Apart from the original use of beeswax by bees for foundation, this type of wax has been traditionally used for the preparation of cosmetics, pharmaceutical products, candles, and other purposes.^131^ Concerning the nutraceutical, pharmacological, and food-processing uses of beeswax, it must be known that there are pharmacopeia standards for BW.^134^ This bee product has been used as a glazing agent on confectionery. Thus, many products of fine bakery are coated with BW. Also, it has been used for the treatment of some types of fruits. Its use is also allowed as a color carrier in food supplements.^135^ In medicine, dated use since the second century BC, BW has been used to coat pills, facilitating ingestion but retarding dissolution. Prepared in a mix with some drugs, it can function as a time-release mechanism.^131^ Additionally, its use has been documented in the treatment of burns, abscesses, wounds, and dental problems.^136,137^

## 7 Conclusion and Future Directions

Bee products (pollen, propolis, bee bread, royal jelly, and beeswax) have been shown to be an important source of bioactive compounds with relevant biological effects. Recently, several studies have started to evaluate their biological activities, such as antimicrobial, anti-inflammatory, antioxidant, anticancer, immune-modulatory, antidiabetic, hypolipidemic, hypotensive, and anti-allergic properties. These pharmacological properties can be undoubtedly ascribed to the multiplicity of the active components that are contained in these complex matrices and that make them emblematic examples of functional foods and sources of bioactive molecules. Despite this, these byproducts are still not considered in the healthcare system because of several factors, including the lack of standardization of their composition, which strongly depends upon several parameters (i.e., geographical area, climatic conditions, plants, methods used for the extraction and analysis, etc.); the scarcity of data regarding the safety, allergy, and toxicity correlated to their use or their therapeutic effectiveness from clinical studies.

Therefore, further studies are strongly needed to (i) standardize the protocols for the extraction and analysis of these food matrices, (ii) assess their content of nutrients and bioactive compounds, (iii) evaluate their bioaccessibility, bioavailability, and metabolism and the influence of the gut microbiota, (iv) measure their safe and toxic dosages, (v) determine their effects in preclinical and clinical studies, (vi) investigate in a deeper way the molecular mechanisms and targets involved in their beneficial effects, and (vii) investigate the possible synergistic/antagonist effects with synthetic drugs. A deeper knowledge and understanding of these honeybee byproducts could be of crucial importance to both promote their use in the general population, for the prevention of the most common pathologies, and discover new pharmaceutical natural products for the treatment of several diseases, in combination with classic therapies.

## Supporting Information Available

Main fat-soluble vitamins (Figure S1) and main polyphenols and other relevant compounds present in bee pollen (Table S1), royal jelly (Table S2), and propolis (Table S3) (PDF)

## Abbreviations Used

BB: bee bread
BP: bee pollen
BW: beeswax
GAE: gallic acid equivalent
IFN-γ: interferon γ
IgE: immunoglobulin E
IgG1: immunoglobulin G1
IL: interleukin
MAPK: mitogen-activated protein kinase
MRJP: major royal jelly protein
MUFA: monounsaturated fatty acid
NF-κB: nuclear factor κ-light-chain-enhancer of activated B cells
PUFA: polyunsaturated fatty acid
RJ: royal jelly
SFA: saturated fatty acid
TNF-α: tumor necrosis factor α
UFA: unsaturated fatty acid
