# Supplementary material for: Bee Products: An Emblematic Example of Underutilized Sources of Bioactive Compounds
Source: J Agric Food Chem. 2022 Jan 2;70(23):6833–48. doi: 10.1021/acs.jafc.1c05822 (PMC9204823; doi:10.1021/acs.jafc.1c05822)
Supplement: Supplementary file 1 — jf1c05822_si_001.pdf [file jf1c05822_si_001.pdf]

Supporting Information

Bee products: an emblematic example of underutilized sources of bioactive compounds

Francesca Giampieri[1,2,3,°], Jose Luis Quiles[4,°], Danila Cianciosi[3], Tamara Yuliett Forbes-Hernández[5], Josè Orantes Bermejo[6], José Miguel Alvarez-Suarez[7,8,*] and Maurizio Battino[3,9,2,*]

[1] *Department of Biochemistry, Faculty of Sciences, King Abdulaziz University, Jeddah, Saudi Arabia*

[2] *Research Group on Food, Nutritional Biochemistry and Health, Universidad Europea del Atlántico, Santander, 39011, Spain*

[3]*Department of Clinical Sciences, Polytechnic University of Marche, Ancona, 60131, Italy*

[4]*Department of Physiology, Institute of Nutrition and Food Technology ''José Mataix'', Biomedical Research Centre, University of Granada, Granada, 1800, Spain*

[5]*Department of Analytical and Food Chemistry, CITACA, CACTI, University of Vigo - Vigo, Spain*

[6]*Apinevada Analytical Laboratory of Bee Products, Barrancos s/n, Lanjarón, 18420 Granada, Spain*

[7]*Departamento de Ingeniería en Alimentos, Colegio de Ciencias e Ingenierías, Universidad San Francisco de Quito, Quito, 170157, Ecuador.*

[8]*King Fahd Medical Research Center, King Abdulaziz University, 21589, Jeddah, Saudi Arabia.*

[9]*International Research Center for Food Nutrition and Safety, Jiangsu University, Zhenjiang, China*

[*]Corresponding authors mails: m.a.battino@univpm.it and jalvarez@usfq.edu.ec.

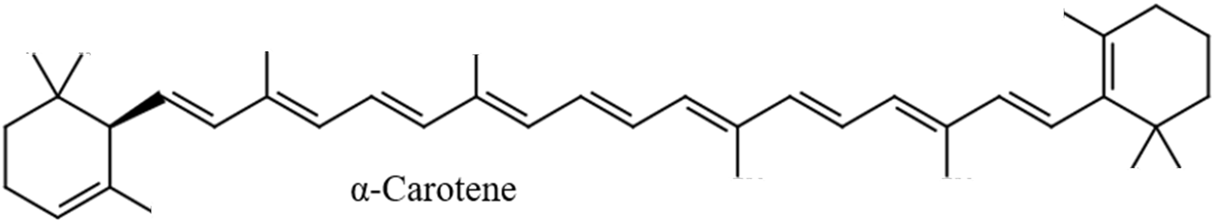
α-Carotene

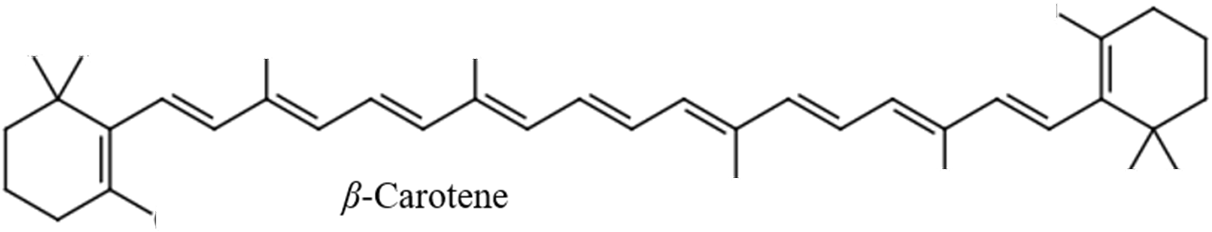
β-Carotene

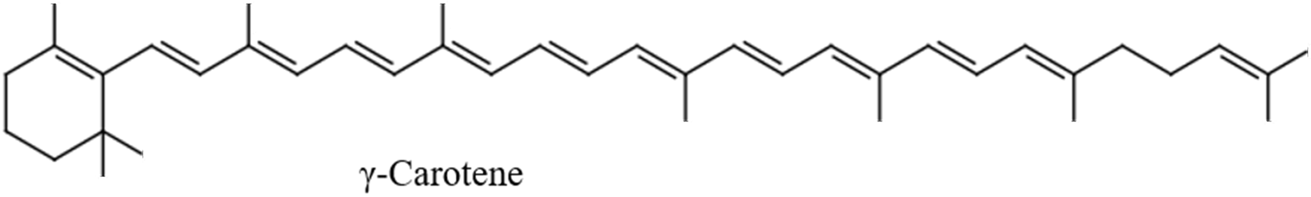
γ-Carotene

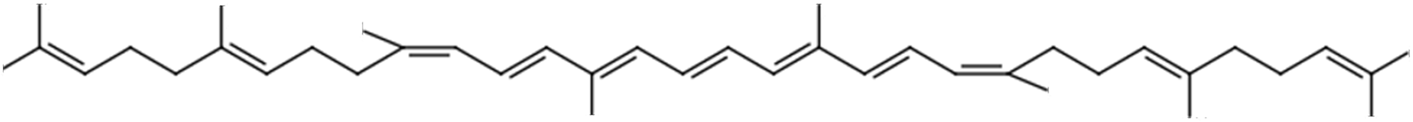
ξ-Carotene

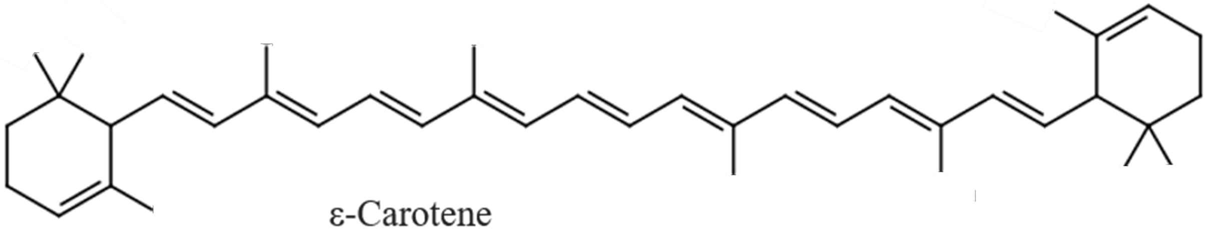
ε-Carotene

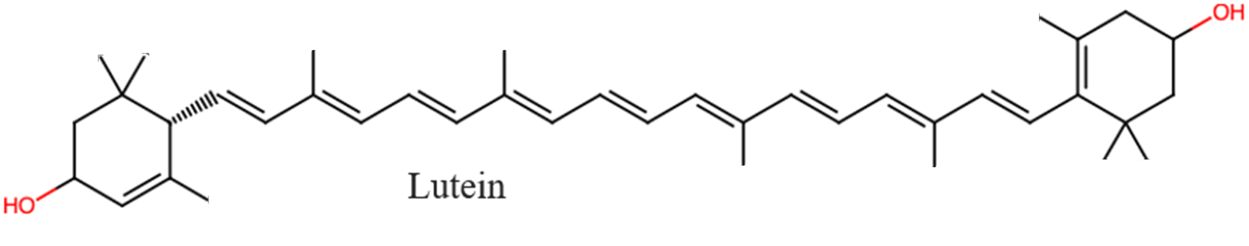

Lutein

Table S1. Main (poly)phenols and other relevant compounds described in bee pollen from different botanical and geographical origins. [9–12]

Chemical Family	Compounds identified
Phenolic acids and derivates	Caffeic acid, *o*-,*p*-coumaric acid, ferulic acid, gallic acid, syringic acid, chlorogenic acid, vanillic acid, coumaroyl quinic acid, protocatechuic acid, tertcinnamic acid, rosmarinic acid, 5-*O*-caffeoylquinic acid, *β*-resorcylic acid, synaptic acid, cinnamic acid, tri-caffeoyl- and caffeoyl-di-p-coumaroyl spermidine derivates, isopimpinellin, hydroxycaffeic acid, benzoic acid, 4- hydroxybenzoic acid, trihydroxycinnamic acid, 3,4-dimethoxycinnamic acid, 2-hydroxycinnamic acid, protocatechuic acid, abscisic acid, trans-cinnamic acid, 3-hydroxytyrosol, isoferulic acid, methoxy-cinnamic acid, tri-p-coumaroyl spermidine, tri-*p*-coumaroyl spermidine (isomer), 1-sinapoyl-2-feruloyl gentiobiose, 5-*O*-caffeoylquinic acid, hydroxycinnamic acid amide derivatives
Flavonoids and derivates	Quercetin, isoquercetin, tricetin, kaempferol myricetin, luteolin, naringenin, selagin, isorhamnetin, epicatechin, hesperetin, rutin, catechin, ampelopsin, eriodictyol, taxifolin, acacetin, genkwanin, apigenin, 3-*O*-methylquercetin, quercetin diglucoside, quercetin-3-*O*-arabinoside, quercetin-3-*O*-rutinoside, quercetin-3-*O*-galactoside, quercetin-3-*O*-rhamnoside, quercetin rutinoside (isomer), quercetin-3-methyl ether, quercetin-*O*-dihexoside, bis-methylated quercetin, dihydroquercetin-3-*O*-rhamnoside, quercetin-*O*-hexosyl-pentoside, quercetin-*O*-rutinoside isomer 1, quercetin-*O*-rutinoside isomer 2, quercetin-*O*-(malonyl)-rutinoside, dihydroquercetin, quercetin-3-*O*-xylosyl-glucuronide, quercetin-3-*O*-sophoroside, quercetin-*O*-(malonyl)hexoside, quercetin-*O*-rhamnoside, quercetin-*O*-acetyl hydroxybenzoyl protocatechuic acid isomer 1, quercetin 3-*O*-galactoside-7-*O*-rhamnoside, quercetin-3-*O*-rhamnosyl-galactoside, quercetin-*O*-acetyl hydroxybenzoyl hydrobenzoic acid isomer 1, quercetin-*O*-acetyl hydroxybenzoyl hydrobenzoic acid isomer 2, 3,7-dimethylquercetin, quercetin-3-O-*β*-D-glucosyl-(2→l)-*β*-glucoside, isorhamnetin-*O*-rutinoside, isorhamnetin-*O*-dihexoside, isorhamnetin-3-*O*-methyl hexuronide, isorhamnetin-3-*O*-rhamnosyl-glucoside, isorhamnetin-3-*O*-rhamnosyl-glucoside (isomer), isorhamnetin-3-*O*-diglucoside, isorhamnetin-3-*O*-rutinoside-7-*O*-glucoside, isorhamnetin-3-*O*-glucoside, isorhamnetin-*O*-(malonyl)hexoside isomer 1, isorhamnetin-*O*-(malonyl)hexoside isomer 2, myricetin-*O*-(malonyl)-hexoside, myricetin-*O*-rutinoside, myricetin-*O*-hexoside, myricetin-3-*O*-glucoside, myricetin-*O*-(malonyl)-rutinoside, hydroxybenzoyl myricetin, myricetin-*O*-dihydroferuloyl, myricetin-*O*-acetyl hydroxybenzoyl protocatechuic acid-isomer 1, myricetin-*O*-acetyl hydroxybenzoyl protocatechuic acid isomer 2, myricetin-O-acetyl hydroxybenzoyl hydrobenzoic acid isomer 2, kaempferol-3-*O*-rhamnosyl-glucoside, kaempferol 3-*O*-sophoroside, kaempferol-3-*O*-glucoside, kaempferol-3-*O*-acetulglucoside, kaempferol 3-*O*-glucuronide, kaempferol-3-*O*-(4″-rhamnosyl)-neohesperidoside, kaempferol-3,4′-di-*O*-*β*-D-glucoside, kaempferol-3-*O*-*β*-D-glucosyl-(2→l)-*β*-D-glucoside, naringin-6′-malonate, naringin-4′-*O*-glucoside, naringenin-7-*O*-glucoside, naringenin hexoxide, luteolin-7-*O*-glucuronide, luteolin-*O*-dihexoside, luteolin-*O*-(malonyl)-hexoside, luteolin-di-*O*-hexosyl-rhamnoside, luteolin-*O*-dihexoside, luteolin-7-*O*-6″-acetylglucoside, apigenin-7-*O*-(6′-malonyl-apiosyl-glucoside), apigenin-6-C-

	glucoside, 4′,5-dihydroxy-7-methoxyflavanone, pinobanksin-5-methylether-3-*O*-acetate
Anthocyanins	Delphinidin-3-*O*-(6’’-p-coumaroyl glucoside), delphinidin-3-*O*-glucoside, delphinidin-3-*O*-glucosylglucoside, delphinidin-3-*O*-rutinoside, cyanidin-3-*O*-xyloside/arabinoside, cyanidin-3-*O*-sophoroside, cyanidin-3-rutinoside, petunidin-3-*O*-arabinoside, petunidin-3-*O*-glucoside, petunidin-3-*O*-rutinoside, petunidin-3-*O*-galactoside, malvidin-3-*O*-rutinoside, pelargonidin-3-*O*-glucoside
Stilbenoids	Resveratrol, *trans*-resveratrol, *cis*-resveratrol, *trans*-piceid, *cis*-piceid
Other relevant compounds	Curcumin, 4-aminobenzoic acid, catechol, caffeine, coumarin, salicylic acid, 2,6-dihydroxy-6-methylbenzaldehyde, 2-formyloxy-1-phenylethanone, anthraquinone derivative, 5-methoxy-7-methyl-1,2-naphthoquinone, 7-hydroxy-1-indanone, methyl benzoate, 2-methyl-5-hydroxybenzofuran,5-methoxy-7-methyl-1,2 naphthoquinone, 1,2,3,4-tetrahydro-2-(2-hydroxy- 3-phenoxypropyl)-6,7-dimethoxyisoquinoline, 1-(2-methoxyphenyl)-9,10-anthracenedione,N′,N″,N″’-tris-p-feruloylspermidine, glucoraphanin, rosmarinic acid dihexoside derivative, N′,N″,N′″-tris-*p*-coumaroylspermidine, pyrogallol, aesculin, tetramethylscutellarein, *O*-dihydroxybenzoyl acetyl malonyl coumaric acid flavonoid derivative

Table S2. Main (poly)phenols and volatiles described in royal jelly from different botanical and geographical origins. [78,90–94]

Chemical Family	Compounds identified
Flavonoids and derivates	Acacetin, apigenin, apigenin-6-C-glucoside, apigenin-7-O-glucoside, apigenin-7-O-neohesperidoside, apigenin-7-O-rutinoside, apigenin-8-C-glucoside, biochanin A, chrysin, coumestrol, daidzein, daidzin, eriocitrin, eriodictyol, fisetin, formononetin, galangin, genistein, genistin, glycitein, glycitin, hesperetin, hesperidin, isosakuranetin, isorhamnetin, isorhamnetin-3-O-glucoside, isorhamnetin-3-O-rutinoside, isosakuranetin, kaempferol, kaempferol-3-O-glucoside, kaempferol-3-O-rutinoside, luteolin, luteolin-4′-O-glucoside, luteolin-6-C-glucoside, luteolin-7-O-glucoside, luteolin-8-C-glucoside, naringenin, narirutin, neoeriocitrin, neohesperidin, phloridzin, pinocembrin, pinobanksin, quercetin 3-O-glucoside, quercetin-3-O-galactoside, quercetin-3-O-rhamnoside, quercetin-3-O-rutinoside, sakuranetin, tamarixetin.
Phenolic and organic acids	Caffeic acid, ferulic acid, p-coumaric acid, 4-hydroxybenzoic acid, 4-hydroxyhydrocinnamic acid, 4-hydroxy-3-methoxyphenylethanol, sinapic acid, syringic acid, dodecanoic acid, octanoic acids and 1,2-benzenedicarboxylic acid.
Volatiles	Ethanol, acetone, methyl acetate, 2-Butanone, ethyl acetate, acetic acid, 3-Methylbutanal, 2-Methylbutanal, 2-Pentanone, 2-Methyl-2-butenal, toluene, butyric acid, hexanal, 2-Heptanone, benzaldehyde, phenol, 2-Methoxyphenol 2-Nonanone, methyl benzoate, octanoic acid, benzoic acid, 2-Methoxy-p-cresol, methyl salicylate, 1-Pentadecene, carbonyls, phenols, aliphatic acids

Table S3. Main (poly)phenols and volatiles described in propolis from different botanical and geographical origins. [110,118,121–124,126]

Chemical Family	Compounds identified
Flavonoids and derivates	Pinocembrin, pinocembrin-5-methyl-ether, pinobanksin, pinobanksin-3-*O*-acetate, pinobanksin-3-*O*-propionate, pinobanksin-2-O-butyrate, pinobanksin-3-*O*-pentanoate, pinobanksin-5-methyl-ether, pinobanksin-5-methyl-ether-3-pentanoate, pinostrobin, naringenin, naringenin-glucoside, sakuranetin, isosakuranetin, liquiritigenin, galangin, chrysin, chrysin-5-methyl-ether, chrysin-6-methyl-ether, tectochrysin, isalpinin, alnustinol, 5-methoxy-3-hydroxyflavanone, hesperetin dimethylated derivates, propolin A, propolin B, propolin E, sigmoidin B, geranylflavanone derivatives, solophenol A, sophoraflavanone A, isosakuranetin, acacetin, catechin, apigenin, izalpinin, quercetin, quercetin 3-*O*-rutinoside, rutin, kaempferol, kaempferol rutinoside, kaempferide, myricetin, fisetin, pectolinarigenin, luteolin, luteolin glucoside, isorhamnetin-3-*O*-rutinoside, macarangin, daidzein, formononetin, pterocarpins, xenognosin B, vestitone, calycosin, vestitol, mucronulatol, neovestitol.
Phenolic acids and derivates	Gallic acid, gentisic acid, chlorogenic acid, cinnamic acid, protocatechuic acid, salicylic acid, vanillic acid, p-coumaric acid, caffeic acid, ferulic acid, caffeic acid phenethyl ester, 4-hydroxybenzoic acid, 4-hydroxyhydrocinnamic acid, and 4-hydroxybenzoic acid-methyl ester, artepillin C, chromenes.
Lignans, stilbenes, coumarins	Syringaresinol, pinoresinol and their dimethyl ether derivatives; scheweinfurthin A, scheweinfurthin B, 5-farnesyl-3'-hydroxyresveratrol, pterostilbene, pinosylvin, O-prenylated tetrahydroxystilbenes and C-prenylated tetrahydroxystilbenes; coumarin, daphnetin and suberosin 1.
Volatiles	β-Eudesmol, guaiol, tricosane, thymol, calamine, cis-verbenol, caryophyllene oxide, bisabolol, eucalyptol, γ-cadinene, α-cadinol, T-cadinol, δ-cadinene, α-pinene, allo-aromadendrene, cedrol, 13-nor-Ambreinolide, 15-nor-Labdan-8-ol, 8-epi-13-nor-Ambreinolide, ambroxide, eicosanol, ethyl linoleate, labd-8-en-15-ol, pinocarvone, eicosane, heneicosane, heptadecane, labd-7-en-15-ol, viridiflorol, nonadecane, pentacosane.
